# Platelet count predicts prognosis in operable non-small cell lung cancer

**DOI:** 10.3892/etm.2013.1003

**Published:** 2013-03-12

**Authors:** DANGFAN YU, BINGJIANG LIU, LIZHEN ZHANG, KAIQI DU

**Affiliations:** 1 Departments of Radiology, Zhejiang Provincial Corps Hospital, Chinese People’s Armed Police Force, Jiaxing, Zhejiang 314000, P.R. China; 2 Thoracic Surgery, Zhejiang Provincial Corps Hospital, Chinese People’s Armed Police Force, Jiaxing, Zhejiang 314000, P.R. China

**Keywords:** non-small cell lung cancer, platelet counts, prognosis

## Abstract

Platelets play a significant role in cancer cell growth, progression and metastasis. However, in non-small cell lung cancer (NSCLC), the association between a patient’s platelet count and prognosis has not previously been fully elucidated. The aim of the present study was to investigate the correlation between platelet count, patients’ characteristics and prognosis in patients with NSCLC. A total of 510 NSCLC patients were enrolled in the present study. The median platelet count in the NSCLC patients was 203×10^9^/l (95% CI, 115–358×10^9^/l). The median platelet count in T3 and T4 patients was significantly higher than that of T1 and T2 patients (median, 263×10^9^/l and 253.5×10^9^/l vs. 199.5×10^9^/l and 196.5×10^9^/l, respectively; P<0.001). The 3-year cumulative overall survival (OS) probability was 75.3% for patients with normal platelet counts and 59.2% for patients with elevated platelet counts. When compared with the patients with normal platelet counts, the patients with elevated platelet counts had an increased risk of disease progression (HR, 1.568; 95% CI, 1.015–2.453). Pre-operative platelet counts are a novel independent prognostic biomarker in operable NSCLC.

## Introduction

Non-small cell lung cancer (NSCLC) is the most common malignant neoplasm worldwide (1), with metastasis and recurrence primarily responsible for mortality. The early identification of metastasis and recurrence provides patients with time to undergo salvage therapy in order to prolong their survival. Radical surgery has been the standard treatment for a number of decades. However, a large number of patients experience disease progression over a short time. Over the past decades, various studies have attempted to identify molecular biomarkers to predict the metastasis or recurrence of NSCLC (2,3). Numerous promising biomarkers have been evaluated as potential prognosis predictors, however, none of these have been demonstrated to be sufficiently effective for clinical use. The majority of these markers are somewhat controversial and inconclusive.

More recently, significant attention has been given to the association between malignancies and coagulation (4,5). A hypercoagulability state is one of the signs of a more aggressive disease, while a thromboembolism is one of the major causes of mortality in cancer patients (6). Several studies have demonstrated that an elevated platelet count correlates with a poor prognosis in numerous types of solid cancer, including colorectal cancer (7,8), esophageal carcinoma (9) and gastric cancer (10,11). A prognostic significance between the platelet count and lung cancer has also been identified (12–17). However, the majority of these studies included small cell lung cancer and the samples were relatively small.

In the present study, a retrospective clinical analysis was designed for a total of 510 operable NSCLC patients to investigate the correlation between platelet count, patients’ characteristics and prognosis.

## Patients and methods

### Patients and treatment

The present study enrolled 510 patients who had been diagnosed with NSCLC between 2006 and 2009 at Zhejiang Provincial Corps Hospital, China. All patients had their diagnoses freshly confirmed and had not had any previous treatment. The patients with the following characteristics were excluded from the present study: patients who had any coexisting or previous cancers other than NSCLC; patients with concomitant diseases suspected of increasing the serum platelet concentration, including severe hypertension, splenic disease and blood coagulation disorders; and patients who had taken aspirin or other acetylsalicylic acid drugs one month prior to the treatment. The study population had a median age of 60 years (range, 37–82 years). The patients comprised of 388 (76.1%) males and 122 (23.9%) females. Approval for the present study was obtained from the institutional review board of the Zhejiang Provincial Corps Hospital. All patients provided informed consent prior to undergoing surgery.

A lobectomy, bilobectomy or pneumonectomy was performed according to the location or size of the lung neoplasm in each patient. Systematic mediastinum lymph node dissection was also performed in each patient. In total, 203 patients were treated with adjuvant platinum-based chemotherapy, adjuvant radiotherapy or a combination of the two. Detailed information about the patient’s characteristics and tumor histopathology were collected retrospectively from the medical records.

### Platelet measurement

A blood sample was obtained by peripheral venous puncture, before breakfast 3 days prior to the surgery. A complete blood count was regularly taken and elevated platelet counts were defined as >300×10^9^/l.

### Follow-up

All patients received a standardized follow-up, occurring at 3-month intervals for two years, at 6-month intervals in the third year and yearly thereafter. An evaluation comprised a physical examination, complete blood count, chest computed tomography (CT), brain magnetic resonance imaging (MRI) and abdominal ultrasound. Local recurrence and distance metastasis were histologically confirmed whenever possible.

### Statistical analysis

The Chi-square test was performed to evaluate the association between the clinicopathological variables and the platelet count. Disease-free survival (DFS) was defined from the date of the definitive surgery to the date of local or distant progression, mortality by any cause or the date of the last follow-up. Overall survival (OS) was calculated as the time from the pulmonary surgery to the time of mortality or censoring. Kaplan-Meier curves were used to estimate the distribution of DFS and OS and a two-sided log-rank test was performed to compare the difference between the survival curves. The Cox proportional hazards model was used with a backward selection method for the univariate and multivariate analyses. All factors with an effect on DFS and OS in the univariate analysis (P≤0.10) were included in the multivariate analysis. All statistical calculations were performed with SPSS 13.0 for Windows (SPSS, Chicago, IL, USA). P<0.05 was considered to indicate a statistically significant difference.

## Results

### Patients’ characteristics

A total of 510 NSCLC patients were enrolled in the present study. The characteristics of the patients are summarized in Table I. According to the criteria of the World Health Organization/International Association for the Study of Lung Cancer (WHO/IASLC) classification of lung tumors, 253 tumors were squamous cell carcinomas, 25 were large cell lung carcinomas and 232 were adenocarcinomas; of these, 29 were well differentiated, 263 were moderately differentiated and 218 were poorly differentiated. There were 354 patients who had smoked at a some time in their lives. In terms of the new IASLC staging system, 234 cases were categorized as stage I, 128 as stage II and 148 as stage III.

### Platelet count and patients’ characteristics

The median platelet count in the NSCLC patients was 203×10^9^/l (95% CI, 115–358×10^9^/l). A total of 449 (88.0%) patients had a platelet count of ≤300×10^9^/l, which was defined as within the normal range. The correlation between the characteristics of the patients and their platelet count is shown in Table I. There was no significant correlation between the platelet count and gender, age, smoking status, tumor histological type or cancer cell differentiation. The median platelet count in the N2 patients was significantly higher than that in the N1 or N0 patients (median, 221×10^9^/l vs. 217×10^9^/l or 192×10^9^/l, respectively; P= 0.001). There was a statistically significant correlation between the platelet count and the T and clinical stages. The median platelet count in the T3 and T4 patients was significantly higher than that in the T1 and T2 patients (median, 263 and 253.5×10^9^/l vs. 199.5 and 196.5×10^9^/l, respectively; P<0.001). When the value of the platelet count was analyzed as a dichotomous variable (elevated and normal platelet count groups), the frequency of the patients with T3 and T4 was higher in the group that had an elevated platelet count. The increased platelet count was also associated with the N and clinical stages (P<0.05).

### Association of platelet count with DFS and OS

**Figure 1:**
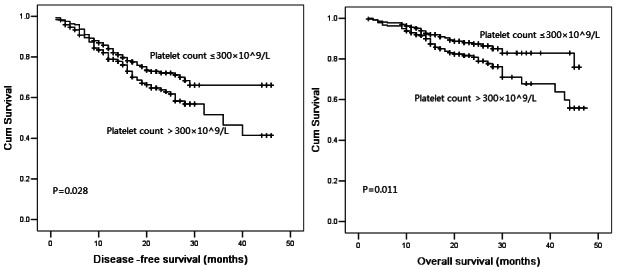
(A) Kaplan-Meier disease-free survival (DFS) curves and (B) overall survival (OS) curves according to platelet count; patients with normal platelet counts showed longer DFS and OS times.

Overall, the 3-year DFS and OS probabilities were 57.3 and 75.7%, respectively. The median DFS period was 34.0 months in the patients with a normal platelet count and 27.4 months in the patients with an elevated platelet count. The 3-year cumulative OS probability was 75.3% for patients with a normal platelet count and 59.2% for patients with an elevated platelet count. The Kaplan-Meier DFS and OS curves of the normal versus elevated platelet counts showed a highly significant separation, as shown in Fig. 1. When stratified by gender, age, smoking status, tumor histological type, tumor differentiation and the T, N and clinical stages in each subgroup, the patients with a normal platelet count had a longer mean DFS time than those with an elevated platelet count. Within the subgroups of age <65 years, female, poor differentiation, T2 stage, N0 stage and clinical stage I in particular, the Kaplan-Meier DFS curves of the normal versus elevated platelet counts showed a significant separation.

In the multivariate survival analysis, the platelet count and patient age, but not the smoking status, tumor differentiation or the T, N and clinical stages, were associated with DFS and OS (Table II).

## Discussion

The results of the present study indicated that the pre-operative platelet count may be used as a biomarker for predicting the outcome in NSCLC. An elevated platelet count was correlated with a worse prognosis in the NSCLC patients. To the best of our knowledge, this is the largest sample study to reveal a correlation between platelet count and the prognosis of operable NSCLC patients.

In the present study, the platelet count was significantly associated with tumor clinical stage and patient outcome. An elevated platelet count was associated with a worse patient outcome. Moreover, multivariate analysis using a Cox proportional hazards model showed that the pre-operative platelet count was an independent prognostic factor in operable NSCLC patients. The patients with an elevated platelet count had a 1.57-fold greater risk of disease progression than those with a normal fibrinogen level. These findings were consistent with a previous study (18), which were based on relatively small sample sizes. Tomita *et al*(18) reported that the pre-operative platelet count was a prognostic factor for resectable NSCLC patients. The 5-year survival probabilities of patients with normal or elevated platelet counts were reported as 28.87 and 63.73%, respectively. The major strengths of the present study are the inclusion of a large population of NSCLC patients, which was used to investigate the prognostic value of the platelet count. The large size of the study may avoid bias and heterogeneity. However, the present study was also a retrospective study and there was insufficient information on post-recurrence treatment, which may have lead to differences in the survival rates. A prospective study is required to determine the prognostic and treatment value of serum fibrinogen.

Platelets play various significant roles in physiological pathways, including homeostasis and inflammation. Also, platelets correlate with the progression of malignancies. The precise reason for the association between an elevated platelet count and a worse outcome for NSCLC remains unknown. Firstly, the increase in platelet count may promote tumor cell growth and angiogenesis. Platelets release various cytokines, including vascular endothelial growth factor (VEGF) and platelet-derived growth factor (PDGF), during blood clotting. The VEGF and PDGF family of proteins has a significant role in regulating angiogenesis. The invasiveness of the cancer cells may be enhanced by the plasma components in stored platelets (19). Additionally, bevacizumab, an inhibitor of VEGF, is able to reduce this promotive effect. Moreover, platelets promote the formation of capillary-like structures by endothelial cells, via integrins mediating cell-cell adhesion (20). Secondly, platelets enhance tumor metastasis by protecting the tumor cells from the host’s immune system. Platelets expressing immunoregulatory proteins, including glucocorticoid-induced TNFR-related (GITR) protein, may protect the cancer cells (21). The inhibition of platelet activation significantly decreases the metastatic potential of tumor cells (22).

In summary, there is evolving evidence that platelet counts are an independent new prognostic biomarker for DFS and OS in operable NSCLC. An assessment of the platelet count should be included in the work up of patients with NSCLC in future prospective trials to confirm its prognostic significance.

## Figures and Tables

**Table I:** Association of platelet count with the parameters of patients with NSCLC.

Parameters	Patients, n (%)	Platelet count				
		Median (5th–95th percentile)	P-value	≤300, n^b^ (%)	>300, n^b^ (%)	P-value
Gender			0.312			0.074
Male	388 (76.1)	204.5 (112.8–373.5)		336 (86.6)	52 (13.4)	
Female	122 (23.9)	198.5 (117.6–352.5)		113 (92.6)	9 (7.4)	
Age (years)			0.263			0.158
<65	353 (69.2)	206.0 (117.0–373.6)		306 (86.7)	47 (13.3)	
≥65	157 (30.8)	193.0 (103.6–357.1)		143 (91.1)	14 (8.9)	
Smoking			0.338			0.094
Never	156 (30.6)	199.0 (115.6–345.5)		143 (91.7)	13 (8.3)	
Smoker	354 (69.4)	205.5 (114.0–378.3)		306 (86.4)	48 (13.6)	
Histological type			0.058			0.127
Squamous cell carcinoma	253 (49.6)	215.0 (117.7–361.0)		216 (85.4)	37 (14.6)	
Adenocarcinoma	232 (45.5)	196.0 (107.0–376.1)		209 (90.1)	23 (9.9)	
Other	25 (4.9)	192.0 (121.0–299.5)		24 (96.0)	1 (4.0)	
Differentiation			0.122			0.959
Well	29 (5.7)	187.0 (78.0–314.5)		26 (89.7)	3 (10.3)	
Moderately	263 (51.6)	201.0 (118.4–333.6)		231 (87.8)	32 (12.2)	
Poorly	218 (42.7)	210.5 (114.8–380.1)		192 (88.1)	26 (11.9)	
T stage			<0.001^a^			<0.001^a^
T1	52 (10.2)	196.5 (96.3–325.8)		47 (90.4)	5 (9.6)	
T2	362 (71.0)	199.5 (117.0–331.4)		329 (90.9)	33 (9.1)	
T3	60 (11.8)	253.5 (127.4–423.5)		43 (71.7)	17 (28.3)	
T4	36 (7.1)	263.0 (148.1–406.1)		30 (83.3)	6 (16.7)	
N stage			0.001^a^			0.001^a^
N0	270 (52.9)	192.0 (107.1–312.5)		251 (93.0)	19 (7.0)	
N1	135 (26.5)	217.0 (110.6–379.2)		114 (84.4)	21 (15.6)	
N2	105 (20.6)	221.0 (117.3–395.3)		84 (80.0)	21 (20.0)	
Clinical stage			<0.001^a^			<0.001^a^
I	234 (45.9)	190.5 (107.5–305.3)		221 (94.4)	13 (5.6)	
II	128 (25.1)	220.5 (113.7–383.8)		107 (83.6)	21 (16.4)	
III	148 (29.0)	225.0 (115.9–387.2)		121 (81.8)	27 (18.2)	

a P<0.05. NSCLC, non-small cell lung cancer;

b ×10^9^ cells/l.

**Table II:** Multivariate analysis of DFS and OS rate.

Parameter	HR	95% CI	P-value
DFS			
Age: <65 vs. ≥65 years	1.416	1.009–1.987	0.044^a^
Smoking: ever vs. never	1.029	0.596–1.777	0.919
T stage: T1 and 2 vs. T3 and 4	1.395	0.892–2.183	0.145
N stage: N0 vs. N1-2	1.113	0.733–1.692	0.615
Clinical stage: I, II vs. III	1.325	0.817–2.147	0.254
Platelet count: ≤300 × 10^9^ vs. >300 × 10^9^ cells/l	1.568	1.015–2.453	0.030^a^
OS			
Age: <65 vs. ≥65 years	1.795	1.170–2.755	0.007^a^
Smoking: ever vs. never	1.367	0.868–2.154	0.178
T stage: T1-2 vs. T3-4	1.157	0.668–2.004	0.602
N stage: N0 vs. N1-2	1.436	0.843–2.444	0.183
Clinical stage: I, II vs. III	1.496	0.834–2.683	0.176
Platelet count: ≤300 × 10^9^ vs. >300 × 10^9^ cells/l	1.689	1.005–2.380	0.017^a^

a P<0.05. DFS, disease-free survival; OS, overall survival; HR, hazard ratio; CI, confidence interval.

## References

[1] Siegel R, Naishadham D, Jemal A (2012). Cancer statistics, 2012. CA Cancer J Clin.

[2] O’Byrne KJ, Gatzemeier U, Bondarenko I (2011). Molecular biomarkers in non-small-cell lung cancer: a retrospective analysis of data from the phase 3 FLEX study. Lancet Oncol.

[3] Douillard JY, Shepherd FA, Hirsh V (2010). Molecular predictors of outcome with gefitinib and docetaxel in previously treated non-small-cell lung cancer: data from the randomized phase III INTEREST trial. J Clin Oncol.

[4] Komurcuoglu B, Ulusoy S, Gayaf M (2011). Prognostic value of plasma D-dimer levels in lung carcinoma. Tumori.

[5] Unsal E, Atalay F, Atikcan S, Yilmaz A (2004). Prognostic significance of hemostatic parameters in patients with lung cancer. Respir Med.

[6] van Doormaal FF, Raskob GE, Davidson BL (2009). Treatment of venous thromboembolism in patients with cancer: subgroup analysis of the Matisse clinical trials. Thromb Haemost.

[7] Monreal M, Fernandez-Llamazares J, Piñol M (1998). Platelet count and survival in patients with colorectal cancer - a preliminary study. Thromb Haemost.

[8] Costantini V, Zacharski LR, Moritz TE, Edwards RL (1990). The platelet count in carcinoma of the lung and colon. Thromb Haemost.

[9] Shimada H, Oohira G, Okazumi S (2004). Thrombocytosis associated with poor prognosis in patients with esophageal carcinoma. J Am Coll Surg.

[10] Ikeda M, Furukawa H, Imamura H (2002). Poor prognosis associated with thrombocytosis in patients with gastric cancer. Ann Surg Oncol.

[11] Hwang SG, Kim KM, Cheong JH (2012). Impact of pretreatment thrombocytosis on blood-borne metastasis and prognosis of gastric cancer. Eur J Surg Oncol.

[12] Gonzalez Barcala FJ, Garcia Prim JM, Moldes Rodriguez M (2010). Platelet count: association with prognosis in lung cancer. Med Oncol.

[13] Gislason T, Nõu E (1985). Sedimentation rate, leucocytes, platelet count and haemoglobin in bronchial carcinoma: an epidemiological study. Eur J Respir Dis.

[14] Engan T, Hannisdal E (1990). Blood analyses as prognostic factors in primary lung cancer. Acta Oncol.

[15] Pedersen LM, Milman N (1996). Prognostic significance of thrombocytosis in patients with primary lung cancer. Eur Respir J.

[16] Aoe K, Hiraki A, Ueoka H (2004). Thrombocytosis as a useful prognostic indicator in patients with lung cancer. Respiration.

[17] Cox G, Walker RA, Andi A (2000). Prognostic significance of platelet and microvessel counts in operable non-small cell lung cancer. Lung Cancer.

[18] Tomita M, Shimizu T, Hara M (2008). Prognostic impact of thrombocytosis in resectable non-small cell lung cancer. Interact Cardiovasc Thorac Surg.

[19] Dineen SP, Roland CL, Toombs JE (2009). The acellular fraction of stored platelets promotes tumor cell invasion. J Surg Res.

[20] Pipili-Synetos E, Papadimitriou E, Maragoudakis ME (1998). Evidence that platelets promote tube formation by endothelial cells on matrigel. Br J Pharmacol.

[21] Placke T, Kopp HG, Salih HR (2011). Modulation of natural killer cell anti-tumor reactivity by platelets. J Innate Immun.

[22] Nieswandt B, Hafner M, Echtenacher B, Männel DN (1999). Lysis of tumor cells by natural killer cells in mice is impeded by platelets. Cancer Res.

